# Diagnostic Performance of Vertical and Sagittal Cephalometric Parameters in Differentiating Skeletal Malocclusion in Saudi Adults: A Cephalometric Study

**DOI:** 10.3390/diagnostics16131977

**Published:** 2026-06-25

**Authors:** Mohammad A. Hamidaddin, Guna Shekhar Madiraju, Faris Yahya I. Asiri, Salem Abdulrahman Albalawi, Abdulelah Abdulrahman Alfalah, Hatim D. Alqurashi

**Affiliations:** 1 Department of Preventive Dental Sciences, College of Dentistry, King Faisal University, Al Ahsa 31982, Saudi Arabia; fasiri@kfu.edu.sa (F.Y.I.A.); halqurashi@kfu.edu.sa (H.D.A.); 2 Dental Intern, College of Dentistry, King Faisal University, Al Ahsa 31982, Saudi Arabia; sal.albalwi@gmail.com (S.A.A.); dralfalahdent@gmail.com (A.A.A.); 3 Department of Orthodontics and Dentofacial Orthopedics, Henry M Goldman School of Dental Medicine, Boston University, Boston, MA 02118, USA

**Keywords:** cephalometrics, malocclusion, FMA, gonial angle, APDI, ROC analysis, orthodontic diagnosis, Saudi adults

## Abstract

**Background/Objective**: This study evaluated the diagnostic performance of vertical growth patterns and mandibular morphology, alongside the anteroposterior dysplasia indicator (APDI), for classifying skeletal malocclusions in a Saudi adult population using cephalometric analysis. **Materials and Methods**: This retrospective cross-sectional discriminatory performance study analyzed 162 archived lateral cephalometric radiographs of Saudi adults aged 18–44 years. The assessed variables included Frankfort-mandibular plane angle (FMA), gonial angle, ANB angle, and APDI. Statistical analysis involved descriptive statistics, ANOVA with post hoc testing, Pearson correlation, logistic regression, and receiver operating characteristic (ROC) curve analysis. **Results**: Significant differences among skeletal classes were observed for all evaluated variables (*p* < 0.05). APDI showed the largest effect size and the highest diagnostic performance, particularly for Class III malocclusion, with excellent discriminatory ability reflected by area under the curve (AUC) values, high sensitivity, and acceptable specificity at optimal cutoff points. FMA showed moderate discriminatory performance, with higher specificity but limited sensitivity, while the gonial angle exhibited comparatively weaker diagnostic performance. In logistic regression analysis, APDI was the only significant independent associated variable of Class II malocclusion. **Conclusions**: Within the ANB-based classification framework used in this study, APDI showed the highest discriminatory performance for skeletal malocclusion classification, supporting its role as a primary sagittal indicator. FMA contributed adjunctive information on vertical skeletal pattern, while the gonial angle showed limited diagnostic value. Combined assessment of sagittal and vertical parameters may improve cephalometric diagnosis.

## 1. Introduction

Skeletal malocclusion is a multifactorial craniofacial condition involving sagittal, vertical, and transverse jaw discrepancies, and skeletal classification support is essential for orthodontic treatment planning and prognosis. Cephalometric analysis remains a cornerstone in orthodontics because it allows clinicians to quantify skeletal relationships and evaluate growth patterns that influence malocclusion development [1,2]. Traditionally, sagittal skeletal relationships have been assessed using angular measurements such as the ANB angle. Although widely used, ANB can be influenced by cranial base length, jaw rotation, and vertical growth pattern, potentially limiting its diagnostic reliability in some cases [3,4,5,6]. Increasing evidence suggests that sagittal discrepancies cannot be interpreted in isolation, as they are closely related to vertical skeletal morphology and overall facial growth pattern [7,8,9,10,11].

Vertical growth pattern, commonly assessed using the Frankfort-mandibular plane angle (FMA), plays a crucial role in craniofacial structure and occlusal relationships [4,12]. Hyperdivergent individuals often exhibit characteristics associated with Class II malocclusion, whereas hypodivergent patterns are more frequently linked to Class III skeletal relationships, supporting an interaction between vertical and sagittal components [7,8,13]. Studies using advanced morphometric or multivariate techniques similarly show that vertical facial divergence significantly influences anteroposterior jaw relationships and treatment-relevant patterns [8,9,10].

Mandibular morphology, particularly the gonial angle, has also been proposed as an indicator of skeletal growth pattern and facial divergence. Larger gonial angles are often associated with vertical growth tendencies and may contribute to sagittal discrepancies, although its independent diagnostic value remains less clearly defined [13,14,15]. To overcome the limitations of single-parameter analysis, composite indices such as the anteroposterior dysplasia indicator (APDI) have been developed. APDI integrates multiple cephalometric components to provide a more comprehensive assessment of sagittal skeletal relationships. Previous studies suggest that composite indices may offer greater diagnostic reliability than conventional angular measurements alone, particularly in borderline cases [1,3,4,5,16].

Despite the availability of multiple cephalometric parameters, many studies have focused on associations rather than diagnostic performance, with limited emphasis on predictive modeling and clinical applicability. Recent advances in orthodontic research emphasize the need for discriminatory performance studies incorporating statistical modeling and ROC analysis to evaluate the real-world utility of cephalometric indicators [1,9,11,16,17]. Furthermore, while traditional cephalometric analysis remains the gold standard for skeletal assessment, recent investigations in Arab populations have begun integrating machine learning approaches to improve orthodontic diagnostic accuracy and classification performance [10,18]. These emerging models depend on reliable foundational cephalometric parameters and population-specific reference standards, underscoring the continued importance of studies evaluating indices such as APDI and vertical growth indicators.

Importantly, craniofacial morphology shows ethnic and population-specific variations, necessitating region-specific investigations. Studies conducted in different populations have shown significant differences in cephalometric norms and skeletal patterns, which may influence diagnostic thresholds and treatment planning [10,19]. Saudi adults with skeletal Class III present distinctive mandibular dimensions and vertical characteristics compared with Class I, differing from patterns reported in other ethnic groups [14]. Longitudinal studies in Saudi children also reveal divergent vertical and sagittal growth trajectories in Class III cases [13]. However, existing Saudi studies have predominantly emphasized descriptive cephalometric characteristics and gender-related differences rather than evaluating diagnostic performance metrics such as sensitivity, specificity, or area under the curve (AUC) values for skeletal classification [20]. Consequently, data specifically addressing the predictive value and discriminatory ability of vertical growth pattern and mandibular morphology in Saudi adult orthodontic patients remain limited.

Given these considerations, there is a need to evaluate the diagnostic contribution of vertical (FMA) and mandibular (gonial angle) parameters in conjunction with sagittal indices such as APDI. Therefore, this study aimed to evaluate the discriminatory ability and comparative performance of FMA and gonial angle, alongside APDI, in differentiating skeletal malocclusion patterns in a Saudi adult population using cephalometric analysis.

## 2. Materials and Methods

### 2.1. Study Design and Setting

This retrospective cross-sectional cephalometric study was conducted using standard digital lateral cephalometric radiographs retrieved from the archives of patients who attended the university dental hospital for orthodontic screening and other diagnostic purposes. The study followed a comparative design based on skeletal classification groups derived from cephalometric analysis. This study was conducted and reported in accordance with the STROBE guidelines.

### 2.2. Inclusion and Exclusion Criteria

Lateral cephalograms obtained between March 2024 and July 2025 were selected using a convenience sampling technique. The Inclusion criteria were Saudi adults aged 18–44 years, availability of good-quality digital lateral cephalometric radiographs with clearly identifiable anatomical landmarks, and patients who presented for orthodontic screening or diagnostic evaluation with no prior orthodontic treatment. Patients who had systemic conditions affecting craniofacial growth and development, craniofacial anomalies or syndromes, significant skeletal deformities, or radiographs of insufficient quality with indistinct anatomical landmarks were excluded.

### 2.3. Ethical Considerations

Ethical approval for the study was obtained from the Institutional Review Board (Ref: KFU-REC-2025-SEP-ETHICS3564). All procedures were conducted in accordance with the ethical principles outlined in the 1964 Declaration of Helsinki. Due to the retrospective nature of the study and the use of anonymized radiographic data, the requirement for informed consent was waived. Patient confidentiality and data privacy were maintained throughout the study.

### 2.4. Study Sample

**Figure 1:**
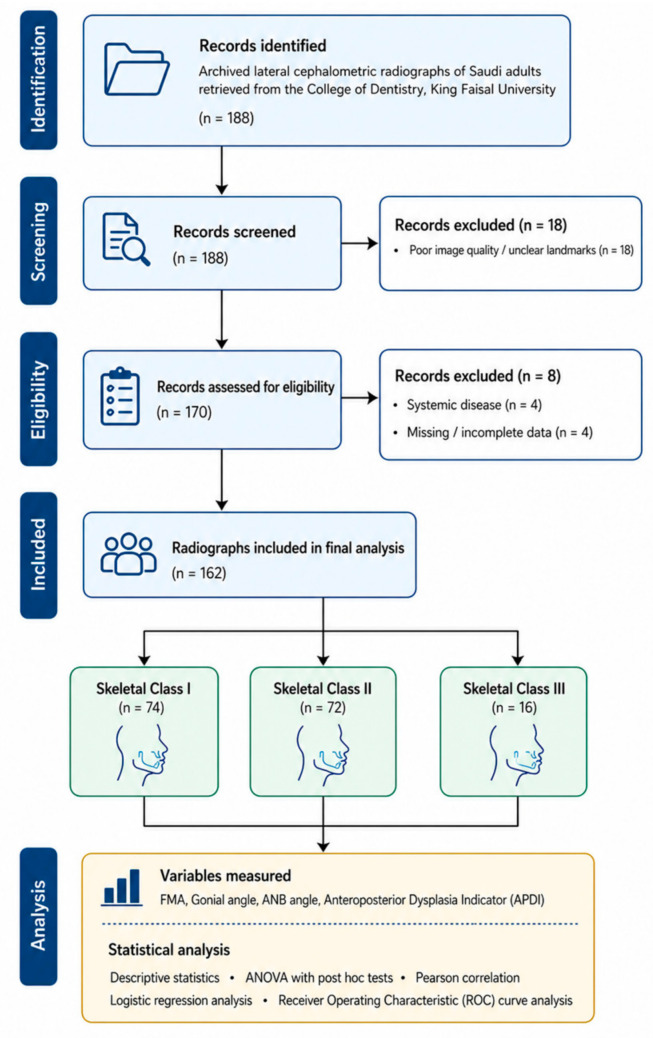
Flowchart of study selection and analysis.

A total of 162 lateral cephalometric radiographs met the eligibility criteria and were included in the present retrospective study. The study selection process, exclusions, eligibility assessment, and final skeletal classification are illustrated in Figure 1. Radiographs of both male and female subjects were included, and only adult patients were selected to ensure completion of craniofacial growth and to enhance the stability and reliability of cephalometric measurements. The sample size for the present study was determined a priori using G*Power software version 3.1.9.7 (Heinrich Heine University Düsseldorf, Düsseldorf, Germany). The calculation was performed assuming a medium effect size (f = 0.25), a significance level of α = 0.05, and a statistical power (1 − β) of 0.80, according to Jacob Cohen criteria for effect size estimation [21]. The minimum required sample size was calculated to be 128 radiographs. However, to account for potential exclusions due to poor radiographic quality, unclear anatomical landmarks, or incomplete records, additional radiographs were included. All lateral cephalometric radiographs were obtained using standardized clinical protocols.

### 2.5. Data Collection and Blinding

The final sample included in the study was selected by a second investigator (GS) according to the predefined criteria. Prior to cephalometric analysis, all radiographs were anonymized by removing identifying patient information, including names, age, gender, and clinical details. Each radiograph was assigned a unique identification code to ensure objective assessment and maintain patient confidentiality. All cephalometric analyses were performed by a single calibrated and experienced orthodontist (HA), who was blinded to patient demographics and clinical background throughout the analysis process in order to minimize observer bias.

### 2.6. Cephalometric Analysis

Cephalometric analysis was performed using WebCeph digital cephalometric software version 2.0.0 (AssembleCircle Corp., Hwaseong-si, Republic of Korea), an artificial intelligence-assisted platform designed for automated cephalometric analysis in orthodontics. Previous studies have reported that the software demonstrates good to excellent agreement with conventional manual cephalometric tracing methods across multiple anatomical landmarks [22]. Anatomical landmarks were manually identified and digitized on each lateral cephalogram, and the resulting AI-assisted measurements generated by the software were visually reviewed and adjusted when necessary to ensure anatomical accuracy. All variables were systematically recorded in a structured dataset for subsequent statistical analysis.

### 2.7. Definition of Variables

**Figure 2:**
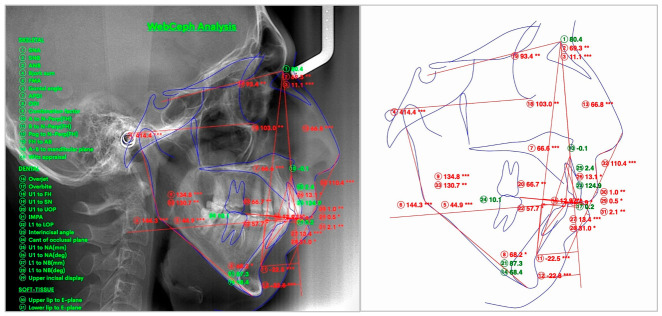
Representative lateral cephalogram of a skeletal Class II adult with superimposed cephalometric analysis from WebCeph software version 2.0.0.

The cephalometric variables evaluated were Frankfort-mandibular plane angle (FMA), defined as the angle between the Frankfort horizontal plane and the mandibular plane, representing vertical growth pattern; gonial angle, defined as the angle formed by the intersection of the posterior and inferior borders of the mandible, reflecting mandibular morphology; ANB angle, used to assess sagittal skeletal relationship; and APDI (Anteroposterior Dysplasia Indicator), a composite cephalometric index used to evaluate sagittal jaw discrepancies (Figure 2).

### 2.8. Skeletal Classification

Classification was based on the ANB angle according to conventional cephalometric criteria. Radiographs were classified into skeletal malocclusion groups using established cutoff values of the ANB angle to define sagittal skeletal relationships: Class I (ANB 0–4°), Class II (ANB > 4°), and Class III (ANB < 0°). This grouping approach was applied for descriptive skeletal stratification within the study sample and not intended as an external diagnostic validation system.

### 2.9. Examiner Calibration and Reliability

To ensure measurement consistency, intra-examiner reliability was assessed. A subset of 20 randomly selected cephalograms were re-analyzed by the same examiner (HA) after a two-week interval under identical conditions. Reliability was evaluated using the intraclass correlation coefficient (ICC) based on a two-way mixed-effects model (absolute agreement, single measures). ICC values were interpreted as follows: less than 0.50 was considered poor, 0.50 to 0.75 moderate, 0.75 to 0.90 good, and 0.90 or above excellent.

Intra-examiner reliability analysis demonstrated excellent measurement reproducibility, with ICC values ranging from 0.88 to 0.95 for all evaluated cephalometric variables (ANB: 0.95, FMA: 0.92, APDI: 0.88, gonial angle: 0.90). Dahlberg error analysis showed minimal method error ranging from 0.28° to 0.45° across variables (ANB: 0.28°, FMA: 0.32°, APDI: 0.39°, gonial angle: 0.45°), indicating minimal measurement variability.

### 2.10. Statistical Analysis

Data analysis was performed using Statistical Package for Social Sciences software (SPSS version 27; IBM Corp., Armonk, NY, USA). Mean and standard deviations were calculated for all variables, and data distribution was assessed using skewness and kurtosis. One-way analysis of variance was used to compare variables across skeletal classes. Homogeneity of variances was assessed using Levene’s test, and post hoc comparisons were performed using Tukey HSD or Games–Howell tests as appropriate. Effect sizes were calculated using partial eta squared (η^2^), with values of 0.01, 0.06, and 0.14 or greater interpreted as small, medium, and large, respectively. Pearson correlation coefficients were used to evaluate relationships among the variables. ANB angle was excluded from inferential group comparisons and regression analyses to avoid incorporation bias, as it formed part of the skeletal classification criteria. However, it was retained in descriptive and correlation analyses to describe sagittal skeletal relationships within the sample. Although APDI incorporates anteroposterior skeletal components that include the A-B plane orientation, it is a composite index derived from multiple craniofacial reference planes and angular relationships rather than a direct replication of the ANB angle. Therefore, APDI was considered methodologically distinct from ANB and suitable for independent evaluation in discriminatory and predictive analyses. Nevertheless, because both APDI and ANB assess sagittal skeletal relationships and share certain craniofacial determinants, some degree of overlap remains. Therefore, the discriminatory performance of APDI should be interpreted with consideration of potential partial incorporation bias.

Binary logistic regression was performed to assess variables associated with skeletal Class II malocclusion. Model performance was evaluated using Nagelkerke R^2^, classification accuracy, and statistical significance of predictors. Multicollinearity was assessed using variance inflation factor (VIF), with values less than 5 considered acceptable. Receiver operating characteristic (ROC) curve analysis was conducted to evaluate the discriminatory performance of cephalometric variables in differentiating skeletal malocclusion groups. Area under the curve (AUC), 95% confidence intervals, and associated *p*-values were reported. A *p*-value less than 0.05 was considered statistically significant. Exact *p*-values were reported where possible; otherwise, thresholds (*p* < 0.001) were used for very small values.

### 2.11. Handling of Missing Data

During eligibility assessment, records with missing or incomplete data were excluded from the study sample. Following this exclusion process, all included cephalometric records were complete, and no missing data were identified in the final dataset. Therefore, no data imputation procedures were required.

## 3. Results

### 3.1. Sample Characteristics

The final study sample comprised 162 subjects, including 71 males (43.8%) and 91 females (56.2%), with a mean age of 30.30 ± 8.19 years. The sample included all three skeletal malocclusion groups, with Class I comprising 74 subjects (45.7%), Class II 72 subjects (44.4%), and Class III 16 subjects (9.9%). No statistically significant association was observed between gender and skeletal malocclusion classification (Pearson χ^2^ = 5.206, *p* = 0.074), indicating that the distribution of skeletal classes did not differ significantly between males and females. This distribution reflects a clinically representative orthodontic population with adequate representation across skeletal patterns to support comparative and discriminatory analyses. The mean values for the study variables are shown in Table 1.

### 3.2. Comparison of Cephalometric Variables Across Skeletal Classes

Significant differences were observed among skeletal classes for all evaluated cephalometric variables (*p* < 0.05), indicating distinct craniofacial characteristics across Class I, Class II, and Class III malocclusion groups (Table 2). Class II subjects exhibited higher FMA and gonial angle values, reflecting a tendency toward vertical growth patterns, whereas Class III subjects showed lower FMA values, suggesting a more horizontal growth pattern. Among the variables evaluated, APDI demonstrated the largest effect size (η^2^ = 0.513), indicating a strong discriminatory ability for differentiating sagittal skeletal relationships within this dataset. FMA showed a large effect size, while gonial angle demonstrated a moderate but still significant effect.

### 3.3. Post Hoc Comparisons

Post hoc analysis using Tukey HSD further clarified pairwise differences between skeletal classes. For gonial angle, Class II subjects demonstrated significantly different values compared with both Class I (mean difference = −3.28, *p* = 0.020) and Class III (mean difference = 4.98, *p* = 0.039), whereas no significant difference was observed between Class I and Class III. These findings suggest that variations in gonial angle were primarily associated with Class II subjects.

For FMA, all pairwise comparisons were statistically significant. Class II showed significantly higher FMA values than Class I (mean difference = −4.05, *p* < 0.001), while Class I exhibited significantly higher values than Class III. Additionally, Class II differed significantly from Class III (mean difference = 6.71, *p* < 0.001), indicating a graded increase in vertical skeletal pattern across the groups. Similarly, both ANB and APDI demonstrated statistically significant differences across all pairwise comparisons between skeletal classes (*p* < 0.001), confirming clear separation among Class I, Class II, and Class III skeletal relationships (Supplementary Table S1).

### 3.4. Correlation Analysis

**Figure 3:**
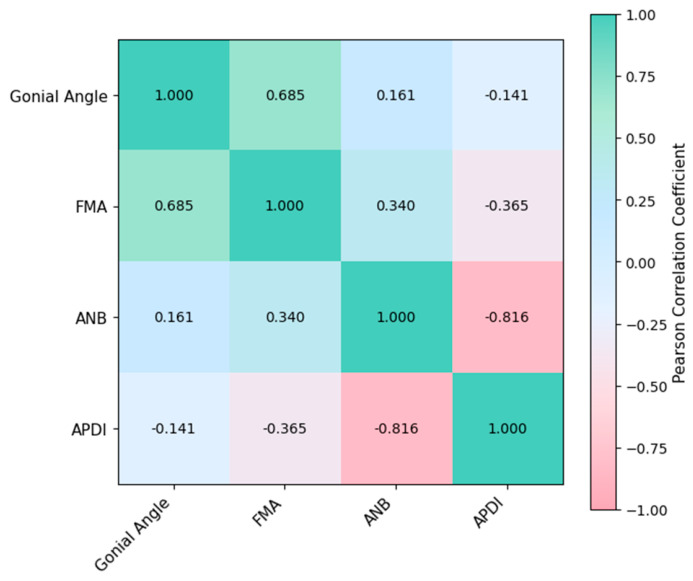
Heat map of Pearson correlation coefficients among gonial angle, FMA, ANB, and APDI, illustrating the strength and direction of associations between vertical and sagittal skeletal parameters.

Pearson correlation analysis revealed significant associations among the evaluated cephalometric variables (Table 3). A strong positive correlation was observed between gonial angle and FMA (r = 0.685, *p* < 0.01), indicating coordinated vertical skeletal development and suggesting that increases in mandibular plane inclination are accompanied by increases in gonial angle. APDI demonstrated a strong negative correlation with ANB (r = −0.816, *p* < 0.01), indicating good agreement with ANB-based assessment of sagittal skeletal discrepancy. In addition, FMA showed a moderate negative correlation with APDI (r = −0.365, *p* < 0.01), suggesting a relationship between vertical growth pattern and sagittal skeletal relationships. A weak positive correlation was found between gonial angle and ANB (r = 0.161, *p* < 0.05), whereas the correlation between gonial angle and APDI was weak and not statistically significant (r = −0.141). These findings indicate that vertical skeletal parameters are more strongly interrelated than their association with sagittal skeletal measurements (Figure 3).

### 3.5. Logistic Regression Analysis for Class II Differentiation

**Figure 4:**
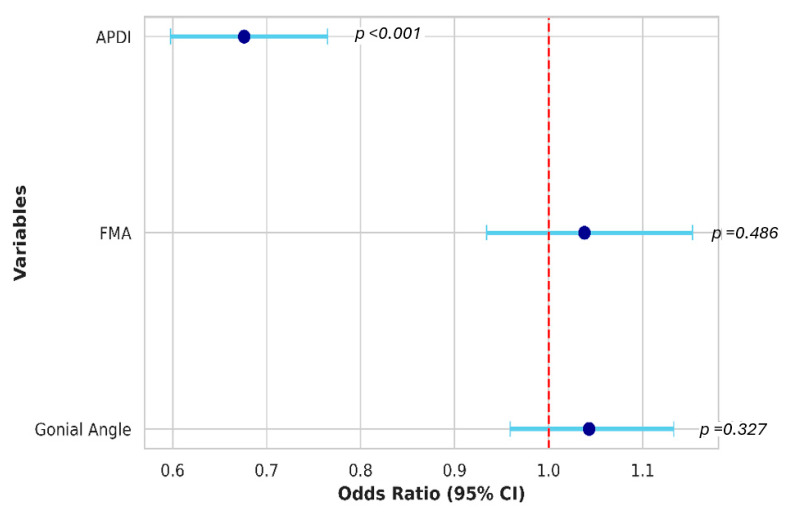
Forest plot of logistic regression analysis for prediction of skeletal Class II malocclusion showing odds ratios and 95% confidence intervals for gonial angle, FMA, and APDI. The dashed red vertical line represents OR = 1.

Details of the logistic regression model for Class II prediction are presented in Table 4. The model demonstrated good explanatory power, with a Nagelkerke R^2^ of 0.606, and achieved an overall classification accuracy of 82.1%. Among the evaluated predictor variables, APDI was identified as the only statistically significant independent variable (*p* < 0.001) (Figure 4). Lower APDI values were significantly associated with an increased likelihood of Class II malocclusion, with each 1° increase in APDI reducing the odds of Class II malocclusion by approximately 32.4% (OR = 0.676; 95% CI: 0.598–0.765). Although FMA and gonial angle showed significant differences in univariate analysis, they did not retain statistical significance in the multivariable model, suggesting that their associations with skeletal classification may partially overlap with sagittal skeletal relationships represented by APDI. ANB was not included in the regression model to avoid incorporation bias, as it was used in skeletal classification.

### 3.6. ROC Curve Analysis

**Figure 5:**
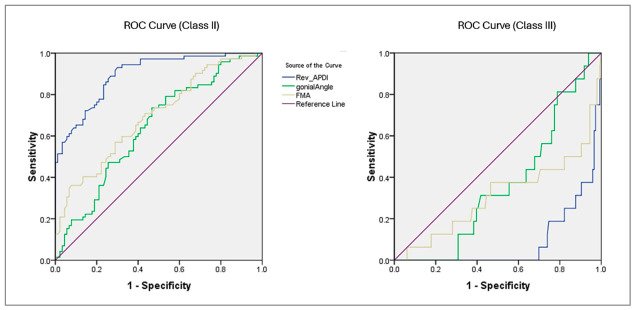
Receiver operating characteristic (ROC) curves demonstrating the discriminatory performance of APDI, FMA, and gonial angle for identifying skeletal Class II and Class III malocclusion.

Receiver operating characteristic (ROC) curve analysis using a one-vs-rest design was performed to evaluate the discriminatory performance of the evaluated cephalometric variables for identifying skeletal Class II and Class III malocclusion patterns (Table 5) (Figure 5) (Supplementary Tables S2 and S3). For skeletal Class II malocclusion, APDI demonstrated excellent discriminatory performance (direction-corrected AUC = 0.896, 95% CI: 0.850–0.943, *p* < 0.001), suggesting good diagnostic potential for differentiating sagittal skeletal discrepancy in this sample. Lower APDI values were associated with Class II skeletal pattern. The optimal APDI cutoff value for Class II discrimination was 84.40°, yielding a sensitivity of 93.1% and specificity of 70.0% (Youden Index = 0.631). FMA demonstrated moderate discriminatory ability (AUC = 0.694, 95% CI: 0.613–0.775, *p* < 0.001), with an optimal cutoff value of 29.73°, corresponding to a sensitivity of 36.1% and specificity of 92.2% (Youden Index = 0.283). Whereas gonial angle showed weak-to-moderate discrimination (AUC = 0.639, 95% CI: 0.554–0.724, *p* = 0.002), suggesting limited independent diagnostic utility owing to its relatively low specificity and modest Youden Index at the optimal threshold.

For skeletal Class III malocclusion, APDI also demonstrated excellent discriminatory performance (AUC = 0.911, 95% CI: 0.854–0.968, *p* < 0.001), indicating good potential for identifying sagittal skeletal discrepancies characteristic of Class III malocclusion, within the study population. The optimal APDI cutoff value for Class III discrimination was 85.78°, with sensitivity and specificity values of 93.8% and 69.9%, respectively (Youden Index = 0.637). In contrast, FMA demonstrated poor inverse discriminatory performance (AUC = 0.312, 95% CI: 0.151–0.473, *p* = 0.014), indicating that lower FMA values were associated with Class III skeletal pattern. FMA demonstrated inverse discriminatory performance for Class III malocclusion (AUC = 0.312), indicating that lower FMA values were associated with Class III skeletal pattern. Due to the inverse ROC direction and limited discriminatory performance, clinically meaningful cutoff thresholds were not considered reliable for standalone diagnostic application. Gonial angle similarly demonstrated poor inverse discrimination (AUC = 0.362, 95% CI: 0.245–0.478, *p* = 0.069), indicating limited effectiveness as an independent diagnostic marker. Because the Class III subgroup contained only 16 subjects, these ROC findings should be interpreted cautiously and considered exploratory until confirmed in larger and more balanced populations.

Overall, among the variables evaluated within the present dataset, APDI showed strongest discriminatory performance for sagittal skeletal, whereas FMA and gonial angle appeared to function primarily as adjunctive indicators related to vertical skeletal morphology rather than primary markers of sagittal discrepancy. The inclusion of optimal cutoff thresholds and associated sensitivity-specificity profiles further supports the potential clinical applicability of APDI and FMA in orthodontic diagnostic screening and skeletal classification.

## 4. Discussion

The present study evaluated the discriminatory performance of vertical growth pattern (FMA) and mandibular morphology (gonial angle), in combination with APDI, in differentiating skeletal malocclusion patterns in a sample of Saudi adults. The findings demonstrate that while vertical and mandibular parameters contribute to craniofacial characterization, APDI showed the strongest discriminatory performance for sagittal skeletal classification, in the present study.

### 4.1. Discriminatory Performance of Evaluated Parameters

A key finding of this study is the very large effect size (η^2^ = 0.513) and excellent discriminatory performance of APDI, in differentiating Class II (AUC = 0.896) skeletal patterns. This supports the concept that composite indices integrating multiple craniofacial components demonstrate stronger discriminatory capability compared with single angular measurements [3,23]. Previous studies have similarly reported improved reliability of APDI compared with conventional sagittal indicators such as ANB [4,23,24]. In the present study, APDI also demonstrated high sensitivity with acceptable specificity at the optimal cutoff thresholds identified using the Youden Index for both Class II and Class III malocclusion patterns, further supporting its clinical utility as a reliable cephalometric indicator for sagittal skeletal discrepancy assessment.

In contrast, FMA showed moderate discriminatory performance for Class II malocclusion (AUC = 0.694), indicating that vertical skeletal pattern is associated with skeletal classification but has limited standalone discriminatory value. This finding is consistent with previous literature describing an association between hyperdivergent vertical pattern and skeletal Class II relationships, often characterized by clockwise mandibular rotation [7,8,12,25,26]. The optimal FMA threshold demonstrated relatively high specificity but limited sensitivity, suggesting that FMA may be more useful as a supportive adjunctive parameter than as an independent diagnostic marker for skeletal classification.

The gonial angle demonstrated comparatively weaker discriminatory performance, indicating a limited independent role in differentiating skeletal malocclusion patterns. Although increased gonial angle has been associated with vertical growth tendency and mandibular morphological changes, its ability to distinguish sagittal skeletal classes appears secondary to composite indices and established sagittal measurements [27]. Similar findings have been reported in previous morphometric and cephalometric studies, where gonial angle variation reflected growth pattern characteristics but did not consistently differentiate sagittal skeletal relationships [8].

### 4.2. Vertical and Sagittal Skeletal Interrelationships

The correlation analysis revealed a strong positive relationship between FMA and gonial angle (r = 0.685), reflecting coordinated vertical skeletal morphology. This finding is consistent with previous evidence suggesting that vertical facial pattern and mandibular plane inclination are associated with mandibular morphology and sagittal skeletal configuration [7,8,25]. Additionally, the moderate negative correlation between FMA and APDI (r = −0.365) indicates an association between vertical growth pattern and sagittal skeletal measurements, consistent with previous reports describing relationships between vertical divergence and anteroposterior jaw relationships [4,23,28].

These findings reinforce the multidimensional nature of craniofacial morphology, where vertical and sagittal components demonstrate interrelated rather than independent behavior in skeletal pattern expression. Previous studies using longitudinal and morphometric approaches have similarly shown that craniofacial growth patterns tend to cluster more strongly according to vertical facial type than sagittal classification, and that vertical facial divergence is associated with variations in anteroposterior skeletal relationships and occlusal characteristics [7,8,25].

However, despite these associations, regression analysis showed that FMA and gonial angle were not significant independent associated variables when APDI was included in the model (*p* = 0.486 and *p* = 0.327, respectively). This suggests that their contribution to skeletal Class II classification is largely shared with sagittal cephalometric parameters rather than reflecting independent associated variables within the evaluated model.

### 4.3. Context Within Cephalometric Literature

Previous research has highlighted limitations of ANB as a standalone sagittal indicator due to its sensitivity to cranial base configuration and mandibular rotation effects [4,16,23,28]. In response to these limitations, composite cephalometric indices such as APDI have been introduced to provide a more stable and comprehensive assessment of sagittal skeletal relationships. The strong association between ANB and APDI observed in this study further supports the reliability of APDI as a sagittal indicator and is consistent with earlier findings that composite indices provide a more stable representation of sagittal skeletal relationships [3,23,24]. It should be noted that sagittal indices such as ANB and APDI are not entirely independent constructs, as both reflect intermaxillary relationships influenced by cranial base and mandibular position. Therefore, their strong association in this study is expected and reflects shared craniofacial determinants rather than redundancy alone. Accordingly, the excellent discriminatory performance observed for APDI in the present study should be interpreted within the context of an ANB-based skeletal classification system. Because both measurements reflect sagittal skeletal relationships, some shared anatomical information may have contributed to the observed ROC performance.

Variability in cephalometric norms across different ethnic and age groups has been well documented, highlighting the need for population-specific validation of cephalometric parameters [4,29]. Several composite sagittal indices, including Yen, Tau, W, Sar, and ABwise have been introduced to improve sagittal assessment and reduce dependence on ANB alone [16,28,30]. Within this framework, the present findings support the applicability of APDI as a useful cephalometric indicator in a Saudi adult population and align with the growing evidence favoring composite cephalometric indices for improved differentiation of skeletal patterns across diverse populations [30,31,32]. APDI integrates both sagittal and vertical skeletal components, which may reduce the influence of mandibular rotation and cranial base variability affecting Wits appraisal and angular Beta measurements. Therefore, APDI may provide a more stable assessment of sagittal discrepancy, particularly in cases with concomitant vertical growth pattern variation.

### 4.4. Clinical Implications and Diagnostic Considerations

From a clinical perspective, the findings of this study have important implications for orthodontic diagnosis and treatment planning. APDI demonstrated superior discriminatory performance among the evaluated variables and may be a useful composite sagittal indicator for differentiating skeletal Class II malocclusion. Its diagnostic utility may be particularly relevant in cases where ANB interpretation is affected by vertical skeletal pattern variation, mandibular rotation, or cranial base configuration [4,28]. In hyperdivergent cases, increased FMA and clockwise mandibular rotation may influence ANB assessment and obscure the true sagittal discrepancy. In such situations, APDI may provide a more stable representation of sagittal skeletal relationships. The observed diagnostic performance further supports its use as a complementary parameter alongside ANB in borderline cases. Although APDI demonstrated excellent discriminatory performance, the optimal cutoff values identified for Class II (84.40°) and Class III (85.78°) were relatively close. This suggests the presence of a transitional diagnostic zone around APDI values of approximately 85°, where classification based solely on APDI may be less reliable. Therefore, APDI should be interpreted alongside complementary cephalometric measurements and clinical findings, particularly in borderline skeletal cases.

FMA demonstrated moderate discriminatory performance and higher values in Class II subjects compared with Class I, supporting its role as an adjunctive indicator of vertical skeletal pattern. It may be particularly useful in borderline Class II cases, high-angle patients, and orthognathic surgical planning, where vertical skeletal divergence can influence mandibular rotation and treatment complexity [25,26,27]. However, its limited sensitivity indicates that FMA should be interpreted in combination with composite sagittal indicators rather than as a standalone diagnostic measure.

The gonial angle showed limited discriminatory performance but may still provide supplementary information regarding mandibular growth pattern and vertical skeletal tendency. Its clinical interpretation is best performed alongside other cephalometric measurements, including FMA and APDI, within a comprehensive diagnostic framework.

The present study utilized conventional two-dimensional lateral cephalograms, which remain widely used in orthodontic diagnosis because of their accessibility and relatively low radiation exposure. However, two-dimensional imaging cannot fully represent the three-dimensional complexity of craniofacial anatomy and may be affected by landmark superimposition, magnification, and projection errors. Advanced imaging modalities such as CBCT provide more comprehensive assessment of skeletal relationships and may improve the precision of diagnostic thresholds. Consequently, the proposed diagnostic thresholds should be interpreted within the context of conventional cephalometric analysis and may require validation using three-dimensional imaging approaches.

Overall, the findings support the combined use of sagittal and vertical parameters to improve diagnostic accuracy in skeletal classification. Population-specific variations in craniofacial morphology should be considered when applying these parameters in clinical practice, particularly in Saudi and regional Arab populations.

### 4.5. Strengths of the Study

This study has several methodological strengths that enhance the robustness and clinical relevance of its findings. A blinded assessment approach was used throughout the analysis to minimize observer and classification bias. AI-assisted cephalometric software was used to support landmark identification and measurement consistency, with visual review and manual adjustment applied where necessary to ensure anatomical accuracy. Rigorous intra-examiner reliability testing further ensured measurement reproducibility. The application of complementary analytical approaches, including effect size estimation, ROC curve analysis, and binary logistic regression, provided a multidimensional evaluation of discriminatory performance extending beyond statistical significance alone. Finally, the focus on a Saudi adult orthodontic population addresses a recognized gap in the regional cephalometric literature, where population-specific data on the discriminatory performance of composite and vertical skeletal parameters remain limited.

### 4.6. Limitations and Future Recommendations

This study has certain limitations that should be considered when interpreting the findings. The retrospective study design may have introduced selection bias, although strict inclusion criteria and anonymization procedures were implemented to minimize its impact. Secondly, the use of ANB-based classification as the grouping reference may introduce partial incorporation bias when evaluating cephalometric variables conceptually related to sagittal skeletal relationships, particularly APDI. Although ANB was excluded from inferential analyses and regression modelling to limit methodological overlap, APDI demonstrated substantial shared variance with ANB, reflecting overlapping craniofacial determinants of sagittal skeletal discrepancy. Consequently, partial incorporation bias cannot be entirely eliminated. Hence, the reported diagnostic performance should be interpreted as reflecting discriminatory ability within the ANB-based classification framework employed in this study rather than definitive evidence of external diagnostic validity.

In addition, the relatively small number of participants with Class III malocclusion, reflecting its lower prevalence within the study population, resulted in substantial imbalance among skeletal groups which may have reduced statistical power and affected the precision and stability of subgroup-specific estimates, particularly ROC-derived diagnostic performance metrics for Class III malocclusion. Consequently, the ROC findings for Class III malocclusion should be considered hypothesis-generating and require external validation in larger cohorts. Furthermore, only intra-examiner reliability was assessed, and inter-examiner reliability could not be evaluated because all measurements were performed by a single calibrated examiner. Although excellent intra-examiner reproducibility was observed, future studies should include multiple calibrated examiners to assess measurement reproducibility and external validity. Finally, the single-center retrospective design may limit the generalizability of the findings; therefore, the results should be interpreted as population-specific and exploratory.

Future research should therefore consider prospective multicenter studies with larger and more balanced samples to improve generalizability and statistical robustness. The incorporation of three-dimensional imaging techniques may provide a more comprehensive evaluation of craniofacial morphology. Furthermore, machine learning–based predictive models integrating multiple cephalometric and clinical variables could enhance discriminatory performance and support more precise skeletal classification. Emerging AI-assisted diagnostic platforms and facial analysis systems may further improve diagnostic workflows through integration of skeletal, dental, and soft-tissue parameters. Additionally, establishing population-specific diagnostic thresholds and cutoff values may further improve the precision and clinical applicability of orthodontic diagnostic tools.

## 5. Conclusions

In conclusion, APDI demonstrated the strongest discriminatory performance, within the ANB-based classification framework used in this study, among the evaluated cephalometric variables in distinguishing skeletal malocclusion patterns in a Saudi adult population, supporting its role as a reliable composite sagittal indicator in orthodontic diagnosis. FMA showed moderate discriminatory value as an adjunctive parameter associated with vertical skeletal pattern in Class II patients, while the gonial angle demonstrated limited independent diagnostic utility and should be interpreted within the overall craniofacial context. Combined assessment of sagittal and vertical parameters may improve diagnostic accuracy, particularly in borderline cases where vertical divergence may influence sagittal interpretation. Overall, these findings support an integrated cephalometric approach to enhance skeletal classification and facilitate individualized orthodontic treatment planning.

## Figures and Tables

**Table 1:** Descriptive Statistics of Study Variables (*n* = 162).

Variable	Minimum	Maximum	Mean ± SD
Age (years)	18.00	44.00	30.30 ± 8.19
Gonial Angle (°)	108.79	152.50	126.40 ± 7.50
FMA (°)	10.63	44.00	24.58 ± 6.21
ANB (°)	−4.77	12.10	3.74 ± 2.83
APDI (°)	68.80	102.10	82.99 ± 5.94

**Table 2:** Comparison of Cephalometric Variables Among Skeletal Classes (ANOVA).

Variable	Class I (Mean ± SD)	Class II (Mean ± SD)	Class III (Mean ± SD)	F-Value	*p*-Value	Effect Size (η^2^)
Gonial Angle (°)	125.10 ± 7.80	128.38 ± 7.24	123.40 ± 4.81	5.159	0.007	0.061 (medium)
FMA (°)	23.04 ± 4.97	27.09 ± 6.19	20.38 ± 7.21	13.649	<0.001	0.147 (large)
APDI (°)	85.42 ± 4.04	78.64 ± 4.14	91.34 ± 4.88	83.758	<0.001	0.513 (very large)

**Table 3:** Pearson correlation matrix showing relationships among gonial angle, FMA, ANB, and APDI.

Variable	Gonial Angle	FMA	ANB	APDI
**Gonial Angle**	1	0.685 **	0.161 *	−0.141
**FMA**	0.685 **	1	0.340 **	−0.365 **
**ANB**	0.161 *	0.340 **	1	−0.816 **
**APDI**	−0.141	−0.365 **	−0.816 **	1

Note: * *p* < 0.05 and ** *p* < 0.01. Note: ANB is a classification-linked variable included for descriptive correlation only.

**Table 4:** Logistic Regression Model for differentiating Class II malocclusion.

Variable	B	SE	*p*-Value	OR (Exp B)	95% CI
Gonial Angle	0.042	0.043	0.327	1.043	0.959–1.133
FMA	0.037	0.054	0.486	1.038	0.934–1.153
APDI	−0.391	0.063	<0.001	0.676	0.598–0.765

Note: OR = odds ratio; CI = confidence interval; SE = standard error.

**Table 5:** Receiver operating characteristic (ROC) analysis and diagnostic performance of cephalometric variables for prediction of skeletal Class II and Class III malocclusion.

Variable	Class II AUC (95% CI)	*p*-Value	Interpretation	Class III AUC (95% CI)	*p*-Value	Interpretation
**FMA (°)**	0.694 (0.613–0.775)	<0.001	Moderate	0.312 (0.151–0.473)	0.014	Poor inverse discrimination
**Gonial** **Angle (°)**	0.639 (0.554–0.724)	0.002	Weak–moderate	0.362 (0.245–0.478)	0.069	Poor inverse discrimination
**APDI (°)**	0.896 (0.850–0.943) *	<0.001	Excellent (direction-corrected)	0.911 (0.854–0.968)	<0.001 (*p* = 0.000)	Excellent

* Direction-corrected AUC indicates that lower APDI values were associated with Class II malocclusion.

## Data Availability

The data presented in this study is contained within the article or Supplementary Material.

## Supplementary Materials

The following supporting information can be downloaded at: https://www.mdpi.com/article/10.3390/diagnostics16131977/s1, Supplementary Table S1: Post Hoc Pairwise Comparison Analysis Using Tukey HSD; Supplementary Table S2: ROC Analysis for Prediction of Skeletal Class II Malocclusion; Supplementary Table S3: ROC Analysis for Prediction of Skeletal Class III Malocclusion.
