# Supplementary material for: Diagnostic Performance of Vertical and Sagittal Cephalometric Parameters in Differentiating Skeletal Malocclusion in Saudi Adults: A Cephalometric Study
Source: Diagnostics (Basel). 2026 Jun 25;16(13):1977. doi: 10.3390/diagnostics16131977 (PMC13359864; doi:10.3390/diagnostics16131977)
Supplement: Supplementary file 1 [file diagnostics-16-01977-s001.zip › diagnostics-4355820-supplementary.pdf]

Supplementary Table S1. Post hoc Pairwise Comparison analysis using Tukey HSD

Multiple Comparisons

Dependent Variable		(I) SkMalOcc	(J) SkMalOcc	Mean Difference (I-J)	Std. Error	Sig.	95% Confidence Interval	
							Lower Bound	Upper Bound
gonialAngle	Tukey HSD	class 1	class 2	-3.28222*	1.21067	.020	-6.1465	-.4179
			class 3	1.69986	2.01640	.677	-3.0707	6.4704
		class 2	class 1	3.28222*	1.21067	.020	.4179	6.1465
			class 3	4.98208*	2.02137	.039	.1998	9.7644
		class 3	class 1	-1.69986	2.01640	.677	-6.4704	3.0707
			class 2	-4.98208*	2.02137	.039	-9.7644	-.1998
	Games-Howell	class 1	class 2	-3.28222*	1.24469	.025	-6.2300	-.3345
			class 3	1.69986	1.50526	.503	-1.9858	5.3855
		class 2	class 1	3.28222*	1.24469	.025	.3345	6.2300
			class 3	4.98208*	1.47397	.005	1.3611	8.6031
		class 3	class 1	-1.69986	1.50526	.503	-5.3855	1.9858
			class 2	-4.98208*	1.47397	.005	-8.6031	-1.3611
FMA	Tukey HSD	class 1	class 2	-4.04579*	.95612	.000	-6.3078	-1.7837
			class 3	2.66664	1.59244	.218	-1.1009	6.4341
		class 2	class 1	4.04579*	.95612	.000	1.7837	6.3078
			class 3	6.71243*	1.59637	.000	2.9356	10.4892
		class 3	class 1	-2.66664	1.59244	.218	-6.4341	1.1009
			class 2	-6.71243*	1.59637	.000	-10.4892	-2.9356
	Games-Howell	class 1	class 2	-4.04579*	.93070	.000	-6.2512	-1.8404
			class 3	2.66664	1.89263	.357	-2.1590	7.4923
		class 2	class 1	4.04579*	.93070	.000	1.8404	6.2512
			class 3	6.71243*	1.94437	.007	1.7971	11.6277
		class 3	class 1	-2.66664	1.89263	.357	-7.4923	2.1590
			class 2	-6.71243*	1.94437	.007	-11.6277	-1.7971
ANB	Tukey HSD	class 1	class 2	-3.73400*	.22945	.000	-4.2768	-3.1912
			class 3	3.90517*	.38215	.000	3.0011	4.8093
		class 2	class 1	3.73400*	.22945	.000	3.1912	4.2768
			class 3	7.63917*	.38309	.000	6.7328	8.5455
		class 3	class 1	-3.90517*	.38215	.000	-4.8093	-3.0011
			class 2	-7.63917*	.38309	.000	-8.5455	-6.7328
	Games-Howell	class 1	class 2	-3.73400*	.21637	.000	-4.2472	-3.2208
			class 3	3.90517*	.52211	.000	2.5637	5.2466

		class 2	class 1	3.73400*	.21637	.000	3.2208	4.2472
			class 3	7.63917*	.53884	.000	6.2698	9.0085
		class 3	class 1	-3.90517*	.52211	.000	-5.2466	-2.5637
			class 2	-7.63917*	.53884	.000	-9.0085	-6.2698
APDI	Tukey HSD	class 1	class 2	6.78283*	.69079	.000	5.1485	8.4172
			class 3	-5.92176*	1.15054	.000	-8.6438	-3.1997
		class 2	class 1	-6.78283*	.69079	.000	-8.4172	-5.1485
			class 3	-12.70458*	1.15337	.000	-15.4333	-9.9759
		class 3	class 1	5.92176*	1.15054	.000	3.1997	8.6438
			class 2	12.70458*	1.15337	.000	9.9759	15.4333
	Games-Howell	class 1	class 2	6.78283*	.67761	.000	5.1781	8.3876
			class 3	-5.92176*	1.30807	.001	-9.2353	-2.6082
		class 2	class 1	-6.78283*	.67761	.000	-8.3876	-5.1781
			class 3	-12.70458*	1.31481	.000	-16.0301	-9.3791
		class 3	class 1	5.92176*	1.30807	.001	2.6082	9.2353
			class 2	12.70458*	1.31481	.000	9.3791	16.0301

*. The mean difference is significant at the 0.05 level.

- Post hoc analysis using Tukey HSD further clarified pairwise differences between skeletal classes.

Supplementary Table S2. ROC Analysis for Prediction of Skeletal Class II Malocclusion

Variable	Class II AUC (95% CI)	p-value	Optimal Cut-off	Sensitivity (%)	Specificity (%)	Youden Index	Interpretation
FMA (°)	0.694 (0.613 – 0.775)	<0.001	29.73°	36.1	92.2	0.283	Moderate
Gonial Angle (°)	0.639 (0.554 – 0.724)	0.002	123.25°	79.2	45.6	0.248	Weak–moderate
APDI (°)	0.896 (0.850 – 0.943)*	<0.001	84.40°	93.1	70.0	0.631	Excellent (direction-corrected)

*Direction-corrected AUC indicates that lower APDI values were associated with Class II malocclusion.

-Receiver operating characteristic (ROC) curve analysis of cephalometric variables for prediction of skeletal Class II malocclusion, including optimal cutoff thresholds derived using Youden Index, with associated sensitivity, specificity, and diagnostic performance metrics.

Supplementary Table S3. ROC Analysis for Prediction of Skeletal Class III Malocclusion.

Variable	Class III AUC (95% CI)	p-value	Optimal Cut-off	Sensitivity (%)	Specificity (%)	Youden Index	Interpretation
FMA (°)	0.312 (0.151 – 0.473)	0.014	Not clinically reliable	-	-	-	Poor inverse discrimination
Gonial Angle (°)	0.362 (0.245 – 0.478)	0.069	Not clinically reliable	-	-	-	Poor inverse discrimination
APDI (°)	0.911 (0.854 – 0.968)	<0.001	85.78°	93.8	69.9	0.637	Excellent

*Direction-corrected AUC indicates that lower APDI values were associated with Class II malocclusion.

-Receiver operating characteristic (ROC) analysis of cephalometric variables for prediction of skeletal Class III malocclusion, including optimal cutoff thresholds and diagnostic accuracy measures.
